# The transcriptome of a complete episode of acute otitis media

**DOI:** 10.1186/s12864-015-1475-7

**Published:** 2015-04-03

**Authors:** Michelle Hernandez, Anke Leichtle, Kwang Pak, Nicholas J Webster, Stephen I Wasserman, Allen F Ryan

**Affiliations:** Divisions of Surgery / Otolaryngology, University of California, San Diego, La Jolla, CA USA; Medicine / Endocrinology, University of California, San Diego, La Jolla, CA USA; Rheumatology, Allergy and Immunology, University of California, San Diego, La Jolla, CA USA; VA San Diego Healthcare System, San Diego, CA USA; Department of Pediatrics, Division of Allergy, Immunology, Rheumatology, and Infectious Diseases, University of North Carolina at Chapel Hill School of Medicine, Chapel Hill, NC USA; Department of Otolaryngology, University of Lübeck, Lübeck, Germany

**Keywords:** DNA microarray, Otitis media, Mouse, Innate immunity, Inflammation, Negative regulators, Gene regulation

## Abstract

**Background:**

Otitis media is the most common disease of childhood, and represents an important health challenge to the 10-15% of children who experience chronic/recurrent middle ear infections. The middle ear undergoes extensive modifications during otitis media, potentially involving changes in the expression of many genes. Expression profiling offers an opportunity to discover novel genes and pathways involved in this common childhood disease.

The middle ears of 320 WBxB6 F1 hybrid mice were inoculated with non-typeable *Haemophilus influenzae* (NTHi) or PBS (sham control). Two independent samples were generated for each time point and condition, from initiation of infection to resolution. RNA was profiled on Affymetrix mouse 430 2.0 whole-genome microarrays.

**Results:**

Approximately 8% of the sampled transcripts defined the signature of acute NTHi-induced otitis media across time. Hierarchical clustering of signal intensities revealed several temporal gene clusters. Network and pathway enrichment analysis of these clusters identified sets of genes involved in activation of the innate immune response, negative regulation of immune response, changes in epithelial and stromal cell markers, and the recruitment/function of neutrophils and macrophages. We also identified key transcriptional regulators related to events in otitis media, which likely determine the expression of these gene clusters. A list of otitis media susceptibility genes, derived from genome-wide association and candidate gene studies, was significantly enriched during the early induction phase and the middle re-modeling phase of otitis but not in the resolution phase. Our results further indicate that positive versus negative regulation of inflammatory processes occur with highly similar kinetics during otitis media, underscoring the importance of anti-inflammatory responses in controlling pathogenesis.

**Conclusions:**

The results characterize the global gene response during otitis media and identify key signaling and transcription factor networks that control the defense of the middle ear against infection. These networks deserve further attention, as dysregulated immune defense and inflammatory responses may contribute to recurrent or chronic otitis in children.

**Electronic supplementary material:**

The online version of this article (doi:10.1186/s12864-015-1475-7) contains supplementary material, which is available to authorized users.

## Background

Otitis media (OM) is the most prevalent disease of childhood in developed countries, accounting for more office visits and drug purchases than any other disease from ages six months to six years [1,2]. Its cost in the U.S. alone is estimated at more than five billion dollars annually in office visits, pharmaceutical agents, surgery and lost productivity [2,3]. Antibiotics provide a generally effective therapy for acute OM (AOM). Since their widespread availability, OM rarely results in a life-threatening disease, as it once did for the proportion of patients who progressed to meningitis. However, in the developing world, OM remains a serious threat to health. The World Health Organization estimates that approximately 30,000 annual deaths can be attributed to OM, and further estimates that half of the world’s burden of permanent, serious hearing loss (>175 million individuals) involves OM [4].

Even in the developed world, there remains a high degree of morbidity associated with both active infection and its more chronic sequelae. Although the long-term impacts are controversial, numerous reports link hearing loss due to persistent OM to deficits in speech perception [5], delayed language acquisition [6] and learning disabilities [7]. Given the cost and potential sequelae of OM, there is a clear need for a better understanding of its pathogenesis in order to develop more effective therapies.

Because the middle ear (ME) mucosa undergoes extensive modification during OM, including the recruitment of nonresident leukocyte species, there is potential for changes in the expression of many genes. The molecular response that occurs in the ME during OM is complex, and provides many opportunities for differences in OM phenotypes among individuals who may express different forms of critical proteins or may have mutations in critical genes. For instance, polymorphisms in several components of the immune system, such as Toll-like receptor 4 (TLR4) [8], Mannose Binding Lectin [9], and pro-inflammatory cytokines such as TNF-alpha (tumor necrosis factor) and Interleukin-1 (IL-1) [10], have been reported to be associated with OM susceptibility, as well as polymorphisms in other genes that impair proper healing after a previous episode of OM [8]. In addition, mouse mutants that exhibit chronic OM have been reported. Mutations in the *Fbxo11* [11] or *Evi1* [12] genes result in chronic OM, perhaps through changes in the development of the ME [11,13].

The role of the mucosa as an important regulator of the immune response has been studied in various disease and therapeutic contexts. A theory regarding the pathogenesis of inflammatory bowel disease is that the intestinal epithelium cannot adequately balance pro- and anti-inflammatory signals in response to enteric bacteria [14]. Mucosal immunity is also being assessed in therapeutics, such as in the development of the intranasal influenzae vaccine [15] and sublingual allergen immunotherapy [16]. The study of the ME mucosa throughout a course of OM may provide insights into the factors that predispose certain individuals to develop its more chronic forms, such as recurrent/chronic OM or OM with effusion.

As so many genes are potentially involved during OM, there is a need to use technologies that can evaluate a large number of gene expression profiles simultaneously in a specific tissue of interest. DNA microarrays offer a broad strategy by which to discover novel genes involved in OM, and to clarify the participation of known genes within complex signaling networks. Previous studies using gene arrays have been performed [17,18] and have yielded valuable information. However, they differed from the current study in several important aspects. These include the use of arrays with limited numbers of genes, characterizing one or two time points after the induction of infection, using heat-killed bacteria, or omitting a control for injection trauma. The present study evaluates the complete kinetics of the ME response to AOM, from initiation of infection to resolution after inoculation with non-typeable *Haemophilus influenzae* (NTHi), one of the most common human pathogens isolated from the ME in OM. Infection with this gram-negative organism characteristically leads to hyperplasia of the ME mucosa and leukocyte infiltration in the ME cavity [19,20]. Analysis of this transcriptome data set highlights the genes that are involved in the acute induction of OM as well as tissue remodeling, bacterial clearance and resolution. It also identifies key pathways that participate in the ME response as well as the interplay between pro- and anti-inflammatory processes that occur in the ME mucosa as it reacts to infection.

## Methods

### Animals

All experiments were performed on naïve, young adult (60–90 days old) WBxB6 F1 hybrid mice (Jackson Labs) during daylight hours, according to National Institutes of Health guidelines on the care and use of laboratory animals and were approved by the Institutional Animal Care and Use Committee of the San Diego VA Medical Center, San Diego, CA. All experiments were conducted in accordance with NIH/PHS policies on the Humane Care and Use of Laboratory Animals. Mice were healthy and were housed under normal SPF conditions in standard rodent boxes with enrichment and free access to food and water. All animals remained healthy during the study. Acute OM in wildtype mice is very similar to that observed in humans [21], but the use of animals allows documentation of the complete course of disease, while the genetic resources available for the mouse are extensive.

### Bacterial strains and culture conditions

*Haemophilus influenzae* strain 3655 (non-typeable, biotype II; NTHi) was used at a concentration of 10^5^ – 10^6^/ml to induce an inflammatory response in the ME. The inocula were prepared as described in Melhus and Ryan, 2003 [21]. Strain 3655 was originally isolated from the ME of child in St. Louis, MO. It is often associated with respiratory tract infections, including otitis media [22]. It was used because we have extensive experience with this strain.

### Surgical procedure

WBxB6 F1 hybrid mice were inoculated bilaterally in the ME with NTHi in a laboratory setting under aseptic conditions. An identical number of control mice were injected with saline. The mice were anesthetized with an intraperitoneal injection of 0.1-0.2 ml per 25–30 g bodyweight of rodent cocktail (13.3 mg/ml Ketamine HCl, 1.3 mg/ml Xylasine and 0.25 mg/ml Acepromazine), which produces deep anesthesia for the required period. The ME bullae were exposed bilaterally by a ventral approach through a vertical midline incision on the neck. To access the ME cavity, the bulla was fenestrated using a 25 gauge syringe needle. Approximately 3–5 μl of NTHi (10^5^-10^6^ NTHi/ml) or endotoxin-free PBS (sham) was injected into the ME cavity of mice and rats. Excess fluid was absorbed with a sterile cotton swab. The wound was closed by replacing the tissue and the skin incision was stapled. After surgery, the animals received a subcutaneous injection of buprenorphine and lactated Ringer’s injection.

### Histology

**Figure 1:**
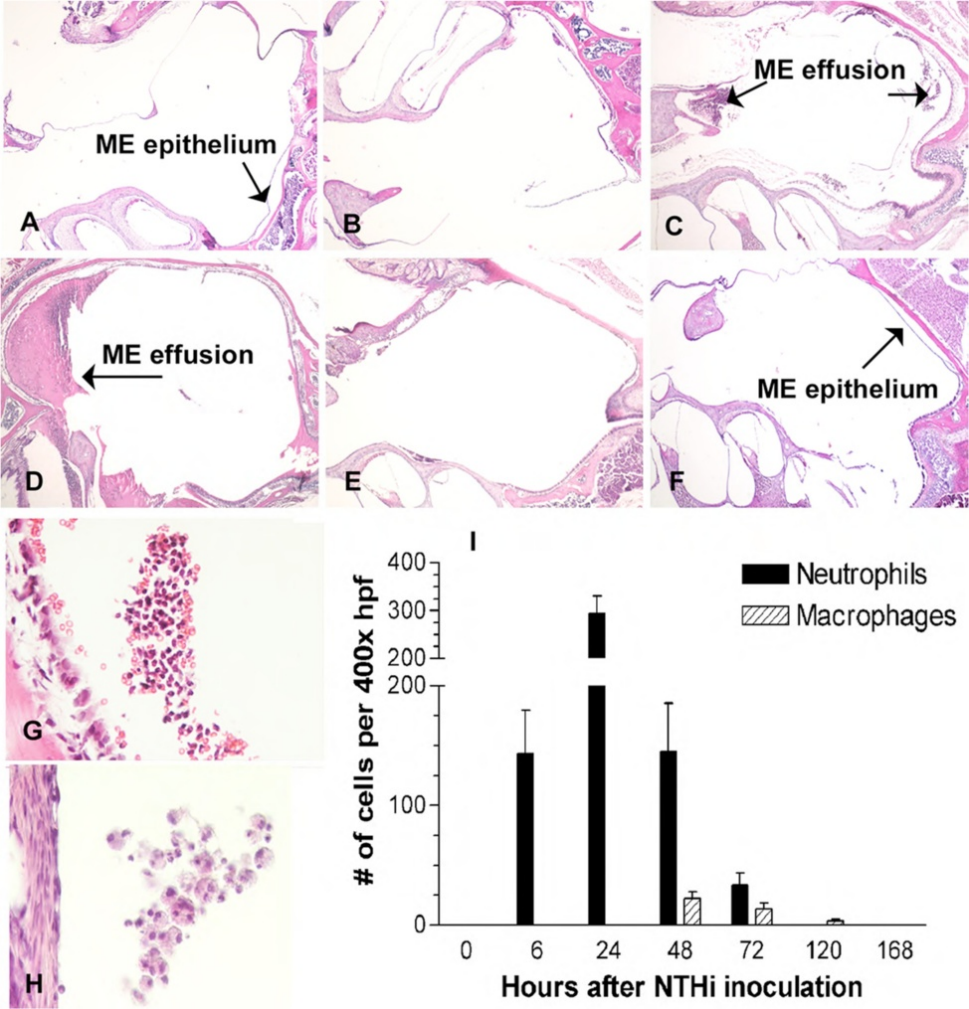
**Otitis Media time course with NTHi. A.** Baseline (0 h) **B.** 6 h **C.** 24 h **D.** 48 h **E.** 72 h **F.** 168 h. The MEs of WBxB6 F1 hybrid mice undergo mucosal hyperplasia with infiltration of inflammatory cells, with a peak of inflammatory changes at 48 hours. The ear resembles its baseline appearance by 168 h (7d). **G.** Neutrophils are evident in the ME cavity by 6 h after inoculation **H.** Macrophages, much less numerous overall than neutrophils, are most prominent at 48 h. **I.** Quantification of neutrophil and macrophage numbers in the ME cavity after NTHi inoculation.

Mice used for histology (3 per time point) were sacrificed under general anesthesia by intracardiac perfusion with PBS followed by 4% Paraformaldehyde (PFA) at 0 hours (0 h, no inoculation), 6 h, 12 h, and 1, 2, 3, 5, 10, 14, and 21 days (21d) after inoculation. For each bacterial preparation, animals from all time points were included. The six MEs were dissected, post fixed (4% PFA) overnight and decalcified (8% EDTA and 4% PFA) for 14d. The decalcified ME bullae of mice were embedded in paraffin and sections were cut at 7 μm. The sections were stained with hematoxylin and eosin (Figure 1). Sections from the same region of each ME, in the largest part of the ME cavity, were digitally recorded and mucosal thickness as well as the percent area of the ME lumen occupied by inflammatory cells were determined at standard locations as described elsewhere [23].

### Inflammatory cell recruitment

The number of neutrophils and macrophages comprising ME cellular infiltrates was assessed by two independent observers, by manually counting these cell types in five randomly selected clusters of cellular ME effusions for each ME in a 400x high power field. The numbers were then computer-averaged.

### DNA microarray

For each array sample, ME mucosae from 20 mice were harvested at each of the following time points after inoculation with either NTHi or PBS: 3 h, 6 h, 24 h (1d), 48 h (2d), 72 h (3d), 120 h (5d), and 168 h (7d). For each bacterial preparation, two groups from widely separated time points were performed (more than two groups could not be performed in a day). Mice were sacrificed, after deep anesthesia as above, by decapitation. Each inoculation time was repeated twice and duplicate, independent samples generated. The tissue was homogenized in TRIzol™ reagent (Invitrogen) and total RNA extracted. Five micrograms of mRNA per treatment per sample were analyzed on two Affymetrix mouse MU430 2.0 gene chips per time point by the Microarray Core at the San Diego Veterans Medical Research Foundation.

**Figure 2:**
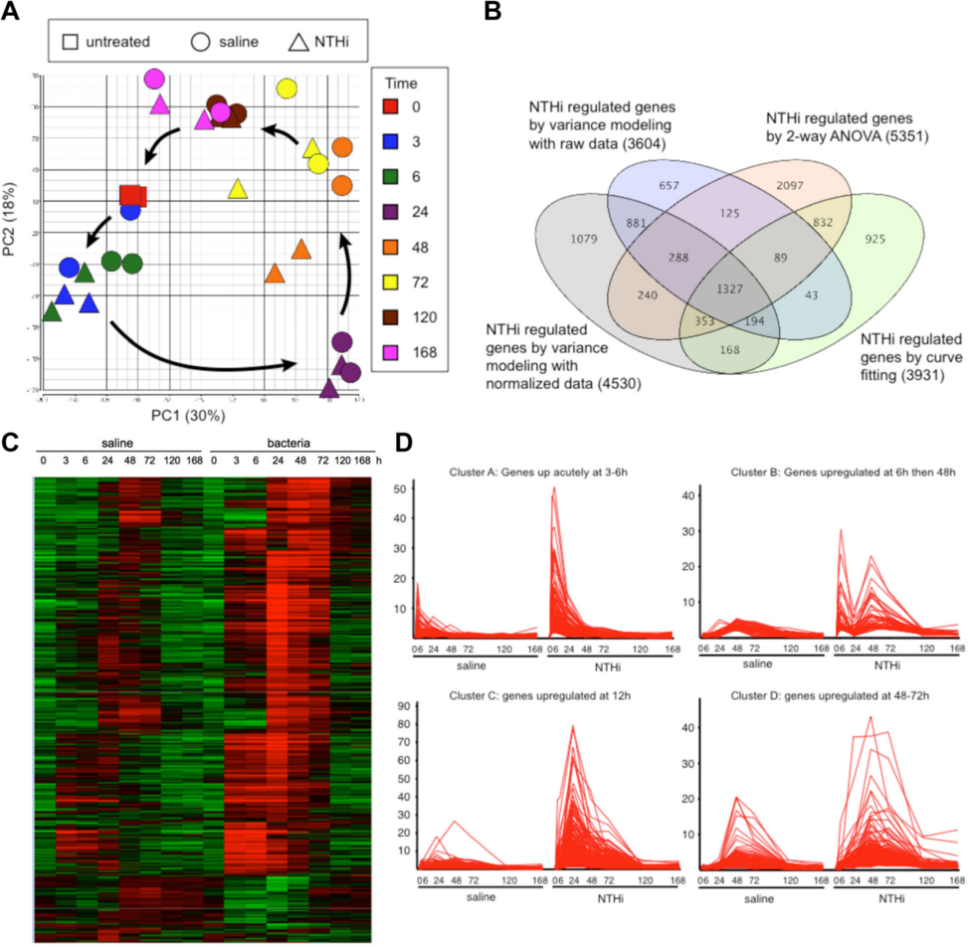
**Transcript signature of AOM with NTHi. A.** Principal component analysis of sample variation. Colors of symbols indicate different times and shapes of symbols indicate different treatments. **B.** 4-way Venn diagram with number of genes differentially expressed with NTHi versus saline treatment as analyzed by four statistical methods. **C.** Heat map for 3657 significant genes identified by the combined statistical analysis, comparing time course of saline treatment to NTHi treatment. The final set of altered genes was defined by combining genes that were significant by 2 statistical methods: variance modeling (raw or normalized data), curve fitting, or 2-way ANOVA). **D.** Expression profiles of four clusters of up-regulated transcripts, showing induction at different times after NTHi inoculation.

Data were analyzed using multiple, independent strategies by an individual blinded to the OM phenotype of each sample. Principal component analysis was performed on the arrays for quality control using RMA (robust multi-array average) normalization [24] in Partek Genomic Solutions (Figure 2A). To identify changes in gene expression, the data were first analyzed using a variance modeling approach. The raw MAS5 expression values were imported into VAMPIRE without prior normalization. This program uses a Bayesian approach to identify altered genes [25]. Statistical analysis by VAMPIRE requires two distinct steps: (1) modeling of the error structure of sample groups and (2) significance testing with *a priori*-defined significance thresholds. VAMPIRE models the existing error structure to distinguish signals from noise and identify the coefficients of expression-dependent and expression-independent variance. These models are then used to identify microarray features that are differentially-expressed between treatment groups. Mice inoculated with NTHi were compared to mice injected with saline at each time point to generate sets of genes that change due to NTHi infection and to eliminate gene changes due to the trauma of the injection. Bonferroni multiple testing correction (MTC, α_Bonf_ < 0.05) was applied to identify only those genes with the most robust changes. The genes altered at each time were combined to give a set of genes differentially regulated between NTHi and PBS. The analysis in VAMPIRE was then repeated using data that had been prior normalized using CORGON [26] and the two datasets compared.

Thirdly, the time course data were analyzed using a curve-fitting algorithm implemented in EDGE. Instead of using a standard statistical comparison of two conditions, one gene at a time, this program implements the Optimal Discovery Procedure to take into account dependent information in the data [27]. This is particularly useful for time course data. The program fits a natural cubic spline to the time course data and then tests whether the fit to the actual data is significantly better than to the data averaged between conditions or to an invariant gene. The time courses following NTHi or PBS inoculation were compared to each other to generate sets of genes whose time course differed significantly between treatments. MTC was applied by false discovery rate (FDR, q < 0.05). Lastly, the data were analyzed by 2-way ANOVA in GeneSpring Ver 12.6 (Agilent, CA) using time and treatment as the two variables. Again, FDR correction was applied to the data. The genes significant for treatment or time treatment were combined to generate a list of NTHi-regulated genes.

The overlap of the four gene sets was compared by Venn diagram for NTHi-regulated genes (Figure 2B). The final list of NTHi-regulated genes was created by combining genes that were positive by >2 statistical approaches (variance modeling, curve fitting or 2-way ANOVA).

This final set of 3657 NTHi-regulated transcripts (Additional file 1: Table S1) was imported into GeneSpring for visualization. The final gene set was subjected to hierarchical clustering using Euclidean distance measurements (Figure 2C). Inspection of the resulting heatmap showed a number of gene clusters with similar expression profiles. The expression profiles of these clusters were generated based on the median-normalized MAS5 data (Figure 2D and Additional file 2: Figure S1).

Lists of genes altered at each time point and treatment generated by VAMPIRE were mapped to gene ontology terms using the program GOby to determine whether any GO classifications were over-represented [25]. MTC (α_Bonf_ < 0.05) was applied to minimize GO terms arising as false positives. Lists of genes in each cluster were mapped to manually curated pathways and processes using MetaCore (GeneGo, St. Joseph, MI). Network analysis was performed on the sets of cluster genes to identify transcription factors (TFs) underlying transcriptional regulation of those genes. TF networks were ranked based on p-values and z-scores in MetaCore. The data were also analyzed for gene set enrichment using gene set enrichment analysis (GSEA) [28]. This approach does not rely on a prior statistical analysis but uses the ranked order of all gene transcripts sampled to look for enrichment in one condition versus another, regardless of whether the expression difference was significant or not. Enrichment of all curated or KEGG pathways (C2) or immunological signature gene sets (C7) in NTHi versus saline was calculated for each time point.

The GSEA was also performed with custom gene sets for epithelial cells and hemaotopoietic cell markers. To assess changes in the epithelium, a new group of gene sets was created by combining all existing gene sets derived from studies of epithelial cells. To assess recruitment of leukocytes and lymphocytes, a published expression dataset on purified human immune cells [29] was reanalyzed in GeneSpring. Two approaches were used to identify cell specific markers for different populations. Initially, the 50–100 most highly expressed genes in each cell-type were selected, then any of these genes that had appreciable expression in other cells were serially subtracted until the final set of selected genes was obtained that had at least 10-fold higher expression in the cell-type of interest. Secondly, a synthetic gene with the desired expression profile was created with high expression in the desired cell and low expression elsewhere, then genes were analyzed for correlation to the synthetic profile. Genes with a correlation coefficient >0.9 were selected. The two lists of cell-enriched genes were combined to give a gene set of cell-type markers. The gene IDs were then translated into mouse genes and the gene set used in a GSEA with the NTHi dataset.

We also created a geneset containing genes that have been associated with OM. This consisted of genes derived from GWAS studies, which were downloaded from the NHGRI website, and a number of candidate genes that have been observed or tested in animal models of OM [30-33].

## Results

### ME responses in AOM

All animals remained healthy throughout the period of the study. Figure 1 illustrates the typical changes seen during a time course of AOM after inoculation with NTHi. The normal tympanic cavity is a relatively quiescent tissue environment in which the ME mucosa and Eustachian tube serve two primary functions: they maintain the gaseous and pressure environment of the ME and maintain a first line of defense against potential infection. Consistent with this role, the ME mucosa of untreated mice consisted of a simple, squamous epithelial monolayer with a minimal stroma. In response to NTHi, the mucosa underwent substantial hyperplasia, converting to a pseudostratified, respiratory epithelium with multiple cell layers and a greatly expanded stroma and vasculature. The hyperplasia peaked at 2-3d after inoculation. This hyperplasia was accompanied by an infiltration of inflammatory cells into the ME cavity as early as 6 h after infection (Figure 1B). Neutrophil numbers peaked in the ME cavity at 24 h after NTHi inoculation, whereas macrophage numbers peaked 48-72 h after inoculation (Figure 1I). While most MEs were culture positive at 1, 2 and 3d post-inoculation, clearance of NTHi was complete by 5d (data not shown). Recovery from OM was characterized by rapid loss of additional cells from the ME mucosa and the ME cavity (Figure 1E-1F), and a return to a relatively simple epithelial morphology by 5-7d after NTHi inoculation.

### Analysis of the AOM transcript signature with NTHi

ME mucosal samples derived from mice inoculated with NTHi were subjected to whole-genome-wide transcript expression profile analysis using oligonucleotide-based DNA microarrays. Principal component analysis of the variance showed that arrays clustered primarily by time with no outliers (Figure 2A). The third principal component of the variance showed separation of the samples by treatment (Additional file 2: Figure S1A). The early time points clustered together as did the late times, with the 5 and 7d time points looking similar to the untreated mice. The greatest variation was seen at 24 h when neutrophil invasion is maximal. Of the 45,000 transcripts represented on these microarrays, 3,657 transcripts were differentially expressed with NTHi inoculation compared to saline injection (Figure 2B, Additional file 1: Table S1). Thus, approximately 8% of the transcripts defines the signature of NTHi-induced AOM in the mouse. Hierarchical clustering of the signal intensities of the individual transcripts showed a number of distinct expression patterns (Figure 2C). Specifically, four clusters of up-regulated gene expression profiles were noted (Figure 2D) and three clusters of down-regulated gene expression profiles were noted (Additional file 2: Figure S1B-D).

### The early induction phase

**Figure 3:**
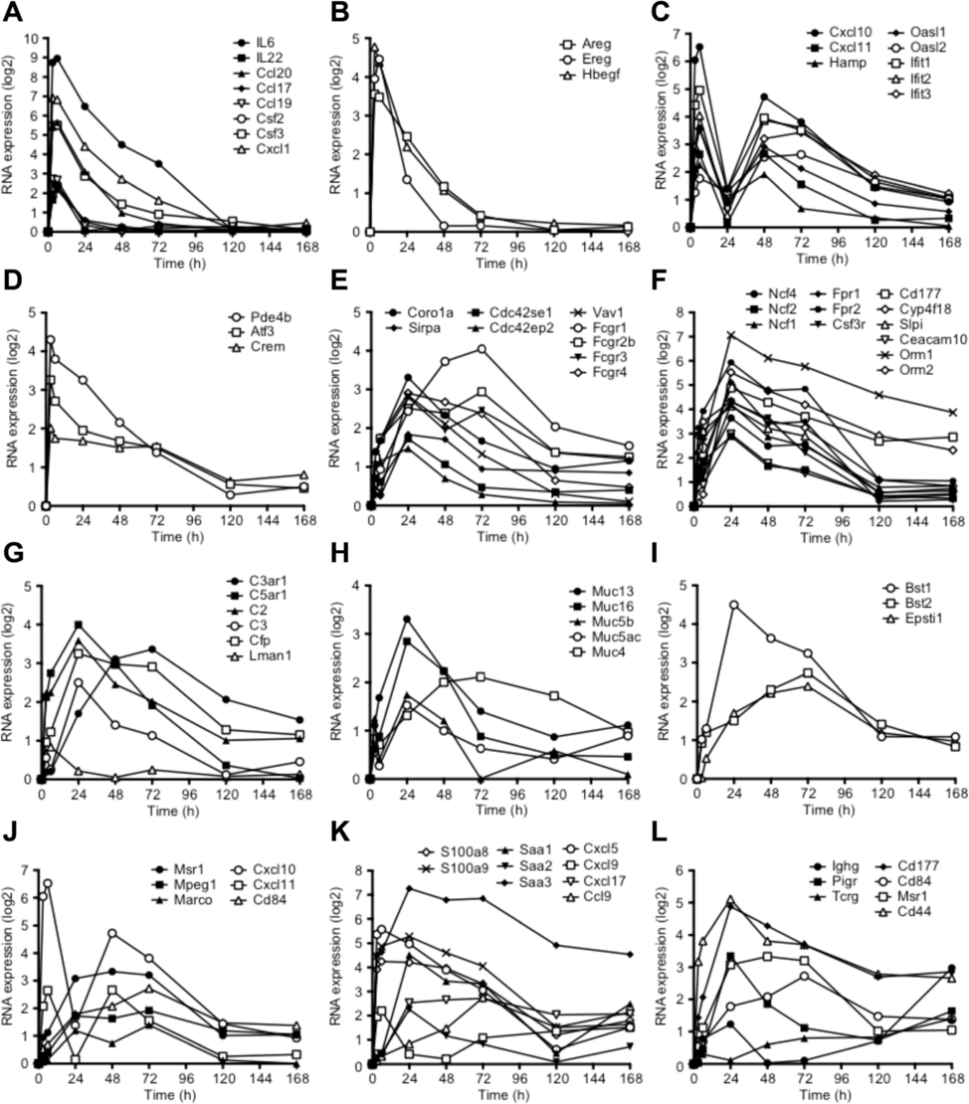
**Gene expression of selected genes. A**-**L.** Expression profiles of selected sets of genes over time after NTHi inoculation plotted as log 2-fold change vs. time 0 (no injection). **A.** Cytokine genes. **B.** EGF family member genes. **C.** Chemokine, anti-bacterial and anti-viral genes. **D**. Genes involved in cAMP signaling. **E**. Phagocytosis genes. **F**. Neutrophil and neutrophil oxidase markers. **G**. Alternative complement genes. **H**. Mucin genes. **I**. Stromal genes. **J**. Macrophage genes. **K**. Antibacterial and cytokine genes that are elevated during the resolution phase. **L**. Lymphocyte and leukocyte markers.

**Figure 4:**
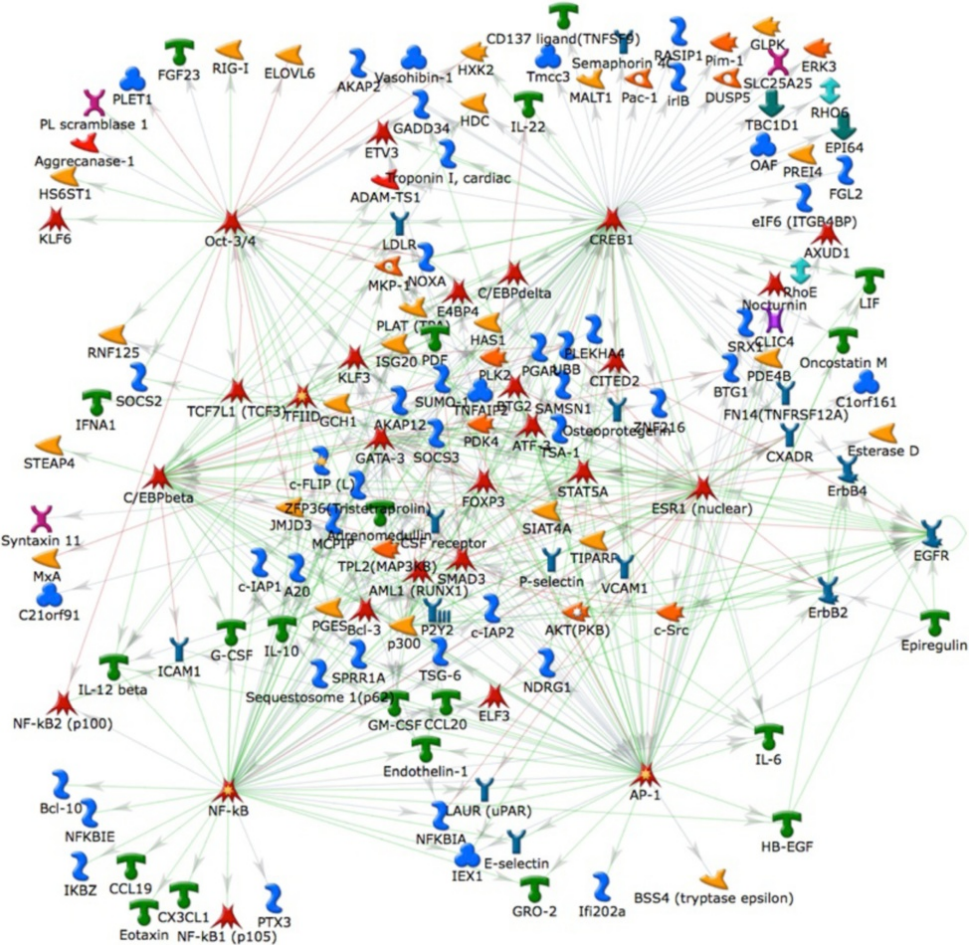
**Dominant TF network analysis of the cluster of genes up-regulated at 3 and 6 hours after NTHi inoculation.** The NFκB and CREB1 networks are most highly represented in the cluster. The lines represent a known stimulatory (green), inhibitory (red) or undefined (grey) interaction between the genes in question. All of the genes in the diagram were up-regulated, with the exception of Oct3/4, TCF7L1, TFIID, CREB1, GATA3, FOXP3, ESR1, p300, AKT (PKB) and c-Src.

Cluster A (Figure 2D) represents the earliest wave of gene expression, peaking 3-6 h after NTHi inoculation with expression extinguished by 24-48 h suggesting that these genes initiate the response to NTHi. This cluster of 259 genes includes *Il6, Il22, Ccl20* (MIP3A)*, Ccl17, Ccl19, Cxcl1* (GRO-1/KC)*, Csf2* (GM-CSF)*, Csf3* (G-CSF) (Figure 3A)*,* the growth factor genes *Hbegf, Ereg* and *Areg* (Figure 3B), and the TNF-induced genes *Tnfaip-2, −3,* and −*6.* GO, pathway and process analysis showed that these genes represent ontology groups and pathways related to immune and defense response, and the response to biotic stimulus and LPS, interleukin, TLR and TNFR signaling, and NFκB and neutrophil activation (Additional file 3: Table S2). CXCL1 is a neutrophil chemoattractant and CSF3 promotes neutrophil survival, proliferation and differentiation, which likely contribute to the appearance of infiltrating neutrophils starting at 6 h [19]. Analysis of transcription factor connectivity showed that NFκB was the most highly connected factor followed by SP1, C/EBPβ, and the AP-1 subunits c-Fos and c-Jun (Additional file 3: Table S2). The largest transcription factor network generated from this cluster contained 105 genes and was focused on NFκB but included CREB1 and C/EBPβ (Figure 4, Additional file 3: Table S2). This may be related to the observation that TLRs can signal via cAMP and JNK in addition to the more well-known NFκB pathway [34]. When signaling pathways were included in the network analysis, the network now included HB-EGF and EREG-mediated EGFR signaling through C/EBPβ, c-Src and AP-1.

Similar enrichment results were found using gene set enrichment analysis of KEGG pathways at 3 and 6 h (Additional file 4: Table S3). GSEA also indicated that 18 of the top 20 enriched pathways at 3-6 h are involved in TLR, cytokine, chemokine and innate immune signaling. The other enriched pathways were JAK/STAT signaling and cell death/apoptosis. Looking specifically at epithelial responses, the NTHi inoculated samples were highly enriched for gene sets in the epithelial response to *H. pylori* and the epithelial to mesenchymal transition (EMT) suggesting that breakdown of the epithelial layer and loss of differentiated phenotype is an early event in AOM. This is supported by the observation that *Zeb1* expression, a marker of EMT [35], is increased at 3-6 h as is the growth factor gene *Hbegf* which promotes EMT in several cell types e.g. [36,37]. We have recently shown that HB-EGF is a potent stimulant of ME mucosal growth *in vitro* [38]. GSEA for transcription factor targets at the 3 h time point indicated that the top 5 gene sets comprised NFκB targets. At 6 h, NFκB targets still scored highly but IRF/IRF2 targets were also in the top 5 enriched sets consistent with induction of interferon expression and signaling at these early times.

The second cluster (Figure 2D, Cluster B) contains 53 genes and is characterized by an early peak of expression at 6 h, then a second peak at 48 h. This cluster is enriched for STAT1/2 and IRF1/7/8 targets and the enriched pathways include interferon and JAK/STAT signaling, anti-viral responses, the inflammasome and leukocyte chemotaxis. Interestingly, the *Stat1* and *Stat2* genes themselves show this pattern of expression. The chemokine genes *Cxcl10* (IP-10), and *Cxcl11* (I-TAC) are present in this cluster as are the antimicrobial peptide gene *Hamp*, and the anti-viral genes *Oasl1, Oasl2, Ifit1, Ifit2,* and *Ifit3* (Figure 3C). These latter genes are expressed by macrophages, so we interpret the biphasic expression to indicate that the early expression is from the epithelial and/or dendritic cells and the later expression due to the infiltrating macrophages that appear 48 h after inoculation.

Four hundred and forty-one genes are down-regulated during this early phase at 6 h after inoculation. Pathway and process enrichment showed that genes involved in ATP metabolism and WNT signaling were altered. Many of these genes were targets for CREB1 and c-MYC The ability of cAMP to repress genes may be related to the observation that *Pde4b, Atf3,* and *Crem1* are induced during this early phase (Figure 3D) to repress CREB activity. GSEA indicates genes involved in fatty acid oxidation and mitochondrial oxidative phosphorylation and protein translation are enriched in the down-regulated genes. Fatty acid oxidation has long been known to be down-regulated by endotoxins in other tissues e.g. [39]. This is consistent with the switch from differentiated oxidative epithelial cells to proliferative glycolytic mesenchymal cells.

### The bacteriocidal phase

A large cluster of 510 genes (Figure 2D, Cluster C) showed peak gene expression 24 h after NTHi inoculation. These genes represent ontology groups and pathways including immune and inflammatory response, chemotaxis, phagocytosis, neutrophil activation, immunological synapse formation, alternative complement activation, cytotoxicity, and ROS production. GSEA at 24 h indicated that genes involved in infection, TLR, chemokine and cytokine signaling are enriched as before, but genes involved in phagocytosis are also enriched, including *Vav1, Sirpa, Cdc42se1, Cdc42ep2, Fcgr2b/3,* and *Coro1a* (Figure 3E)*.* This cluster also contains markers for neutrophils (*Csf3r, Fpr2, Cd177, Orm1, Orm2)* and for the neutrophil NADPH oxidase subunits *Ncf1, Ncf2* and *Ncf4* that mediate superoxide burst production (Figure 3F). This coincides with the maximal infiltration of neutrophils that is seen histologically. Also elevated are the genes *S110a8* and *S100a9* that facilitate NADPH oxidase complex formation and activation, and that are prominently expressed in neutrophils*.* Genes involved in alternative activation of the complement pathway (*C5ar1, C2, Cfp, C3, C1qbp,* and *Lman1)* (Figure 3G) and mucin production *(Muc5ac, Muc5b, Muc13,* and *Muc16)* are also induced (Figure 3H), indicating ongoing bacteriocidal activity*.* Stromal genes such as *Bst1/2* and *Epsti1* are elevated consistent with the proliferation of stromal cells (Figure 3I). Interactome connectivity analysis of the transcription factors driving this expression pattern showed that PU.1 was the most over-connected (Additional file 3: Table S2; Additional file 5: Figure S2), along with SP1 and STAT3. The largest transcription factor networks, however, were derived from CREB, SP1, and c-MYC indicating that quantitatively these TFs have more targets, but that their targets are not as enriched as those for PU.1 (Additional file 3: Table S2).

There is also a cluster of 358 genes that are down-regulated 24 h after NTHi inoculation (Additional file 2: Figure S1, Cluster F). These genes represent ontology groups related to microtubule-based movement, ciliary morphogenesis, and cell projection organization consistent with de-differentiation and possibly the loss of ciliated epithelial cells. We have previously reported that induced expression of one of these genes, *Ecrg4*, decreases the growth of infected ME mucosa, and reduces leukocyte infiltration of the ME during OM [40]. GSEA showed enrichment of genes involved in fatty acid and amino acid metabolism and oxidative phosphorylation similar to the genes down-regulated at 6 h, but also showed decreases in drug and xenobiotic metabolism, extracellular matrix interaction, tight junction formation, and hedgehog and TGFβ signaling in agreement with the progressive epithelial-to-mesenchymal transformation.

A subsequent peak of gene expression occurs at 48-72 h (Figure 2D, Cluster D). This cluster contains 164 genes and consisted of genes involved in the alternative complement pathway, CCL2 signaling, chemotaxis, phagocytosis that are significantly up-regulated at this relatively late stage. This cluster included macrophage specific markers such as *Marco, Msr1, Mpeg1,* and *Cd84,* indicating the infiltration of macrophages at this time point (Figure 3J). These responses are consistent with tissue remodeling and the initial stages of cognate immunity. The most significant TF was the macrophage specific factor PU.1. A cluster of 98 genes showed repressed expression between 24-48 h. Within this gene set, transcripts representing morphogenesis and development predominated.

### The resolution phase

By 5 to 7d after NTHi inoculation, the ME mucosa has remodeled mostly back to baseline, and the inflammatory cell infiltrate has disappeared. While most genes had returned to pre-inoculation levels, 164 genes remained significantly up-regulated 7d after NTHi inoculation. Amongst these transcripts, the orosomucoid genes *Orm1/2* (Figure 3F), serum amyloid genes *Saa1/2/3*, and the *S100a8/9* genes (Figure 3K) exhibited continued expression compared to saline, suggesting ongoing anti-bacterial surveillance. Markers such as *Msr1, CD177, Cd84, Ccl9* and *CXCL5/9/17* are still elevated indicating the continued presence of leukocytes and, interestingly, lymphocyte marker genes such as *CD3, Pigr, Igh,* and *Tcrg* are observed, indicating the induction of cognate immunity (Figure 3L). In contrast, genes encoding innate immune receptors and signaling molecules were not represented, consistent with a lack of any type of “memory” that could be associated with enhanced long-term expression of genes in the innate immune system.

### Participation of cell types during AOM with NTHi

Additional analytical tools were used to track the appearance of specific leukocytes during the course of AOM. In particular, GSEA was applied using gene sets derived from human hematopoetic cell profiling and other immunological signature data sets. This analysis provided an analysis of cell infiltration between NTHi versus saline injection by the simultaneous analyzing of numerous sets of genes typically expressed in leukocyte subsets not just known cellular marker genes. After 3-6 h of inoculation, the top 20 enriched gene sets are all derived from LPS-stimulated monocytes, macrophages or DCs (Additional file 4: Table S3). Since macrophages are typically not observed in the ME at this time [19], this suggests the presence and response of dendritic cells which can be difficult to identify in H & E sections. At 24 h, thirteen of the top 20 gene sets are monocyte derived, but gene sets from T-cells, neutrophils and eosinophils are also enriched. This confirms the data above regarding neutrophil-specific markers. While we do not see eosinophil and T-cell markers in our cluster analysis, this may reflect that the genes did not reach significance due to the multiple testing correction, or they did not appear in any particular cluster. Monocyte gene sets are again predominant at 48-72 h in agreement with the appearance of macrophage specific markers, but gene sets derived from the response to viral infections or vaccine treatment are also enriched. At later times, the top enriched gene sets are derived from vaccine-stimulated, virally-infected and lupus derived peripheral blood mononuclear cells and T-cell derived gene sets. These enriched T-cell gene sets indicate that lymphocytes involved in the adaptive immune response remain in the ME mucosa after resolution of the acute infection, however the presence of these lymphocytes is not easily observed by routine H & E staining of the ME.

### Both pro- and anti-inflammatory cytokine and cytokine signaling genes are up-regulated rapidly in response to NTHi

As pro-inflammatory cytokine signaling ontology groups are represented both at the early induction and middle bacteriocidal stages of AOM, we evaluated the expression profile of cytokine signaling genes during NTHi infection. Figure 3A shows that NTHi up-regulates expression of IL-6 by approximately 500-fold at 3-6 h after inoculation. The IL-6 peak declines, followed by significant increases in other general pro-inflammatory cytokines such as IL-1 beta (60X) and TNF-alpha (130X) peaking at 24 h after NTHi inoculation. Pro-inflammatory cytokine expression was accompanied by up-regulation in anti-inflammatory cytokines such as IL-1 receptor antagonist (130X), which competes for binding to the IL-1 receptor, and IL-10 (9X at 3-6 h) (Additional file 5: Figure S2).

**Figure 5:**
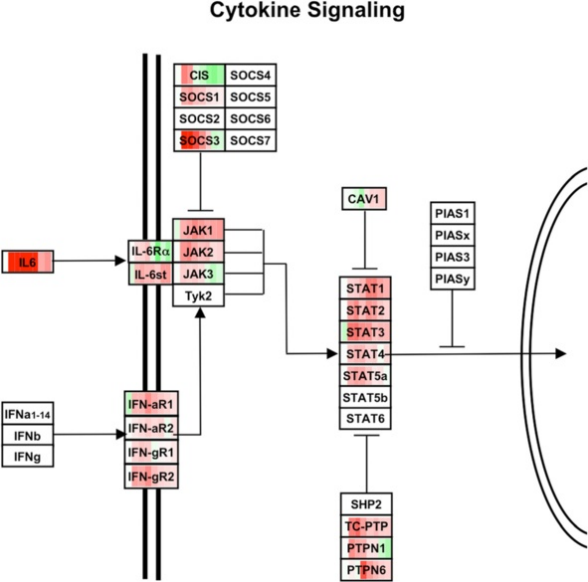
**Regulation of genes in the JAK/STAT signaling pathway.** Diagrammatic representation of responses for genes involved in JAK/STAT signaling. For each gene, expression has been represented relative to 0 h across the course of AOM, from 3 h to 7d; with white representing no change, red: up-regulation and green: down-regulation. The degree of regulation is indicated by color intensity. Both proinflammatory and anti-inflammatory signaling genes are regulated with similar kinetics. An exception is the STAT inhibitor CAV1, which is down-regulated early in AOM.

Regulation of genes in the downstream JAK/STAT pathway was visualized on a pathway diagram, in which expression relative to pre-inoculation levels were color-coded across time (Figure 5). In general, both pro- and anti-inflammatory elements within the network were activated with very similar kinetics, characterized by early up-regulation. An exception was CAV1, a direct negative regulator of STATs, which was down-regulated early, then up-regulated later in OM.

### Innate immune signaling genes are up-regulated in AOM with NTHi

**Figure 6:**
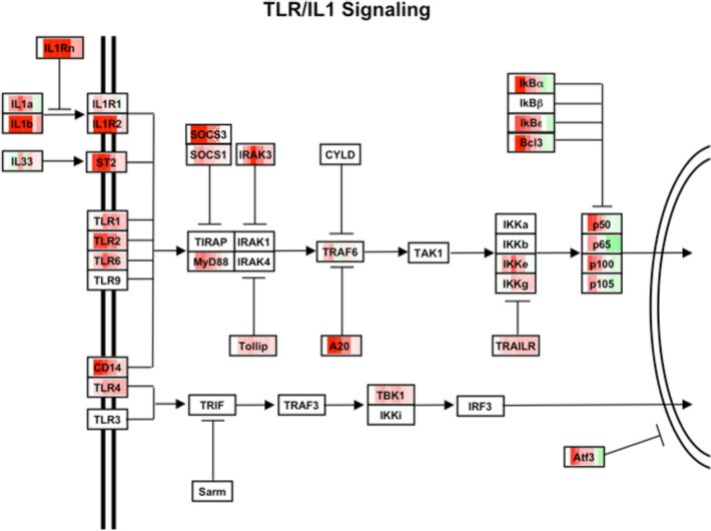
**Regulation of genes in IL1/TLR signaling pathways.** Diagrammatic representation of responses for genes involved in IL-1 and TLR signaling during NTHi-induced AOM. Gene regulation indicated as in Figure 5. Pro-inflammatory genes within the direct signaling pathway and anti-inflammatory regulatory genes are expressed with similar kinetics.

The kinetics of the pro-inflammatory cytokine profile seen in the NTHi-treated groups suggested that signaling through TLRs was important in initiating the inflammatory response in AOM. These changes are visualized on the TLR/IL1 signaling pathway diagram as for the JAK/STAT pathway before (Figure 6). Although none of the TLRs was up-regulated significantly with sham inoculation, several were up-regulated with NTHi (Additional file 5: Figure S2). TLR2 was strongly induced in the NTHi treated group, with significant increases occurring 3 h after inoculation and peaking at 24 h (40X). TLR13 was induced 5-X in NTHi treated groups compared to sham at 24 h after NTHi inoculation, though the function of this murine-specific TLR has not been defined to date. While TLR4 was not significantly up-regulated, expression of the TLR4 co-receptor CD14 was strongly enhanced by NTHi (11X at 24 h). NOD-like receptor (NLR) family members were also significant after Bonferroni correction. NLRP3, also known as NALP3 and CIAS, is a member of the caspase-1 inflammasome leading to pro-IL-1 beta and pro-IL-18 processing. NLRP3 was modestly but significantly up-regulated (3-4X) at 6 and 24 h after NTHi treatment compared to sham. Another element of the inflammasome, ASC (also known as PyCard) was significantly up-regulated (3X) 24 h after NTHi inoculation. We have recently shown that mice deficient in ASC exhibit dramatically reduced IL-1 beta processing and delayed bacterial clearance from the ME during OM [41].

### Positive and negative regulators of innate immune signaling are up-regulated by NTHi with similar kinetics

The pro-inflammatory burst noted above is also accompanied by induction of negative regulators of TLR- and NOD-associated intracellular signaling that serve to dampen the induction of cytokine production. As illustrated in Figure 6, IRAK3, a negative regulator of TLR signaling which prevents the dissociation of IRAK-1 and IRAK-4 from MyD88 [42], was induced 6 h after NTHi inoculation, peaking 24 h (35X) after inoculation and remaining elevated through 120 h. Similarly, we see induction of inhibitor of kappa-B kinase *Ikbke* and nuclear factor of kappa light polypeptide gene enhancer in B cell inhibitors *Nfkbia/b/d/e* that dampen NFκB signaling. Suppressor of cytokine signaling 1 (SOCS1, 10-15X 3-24 h) and SOCS3 (5X, 3-6 h) were also evaluated. NLRP12, a negative regulator of TLR signaling via inhibition of NFκB activation by either canonical or non-canonical signal transduction pathways [43], was significantly up-regulated compared to sham treatment at 3 and 24 h after NTHi treatment (5-14X).

## Discussion

As a survey tool, DNA microarray analysis can provide simultaneous data on the expression of essentially all mouse genes during AOM. Using this approach, our results identify discreet waves of genes that are activated at distinct times throughout a course of AOM induced by NTHi.

**Figure 7:**
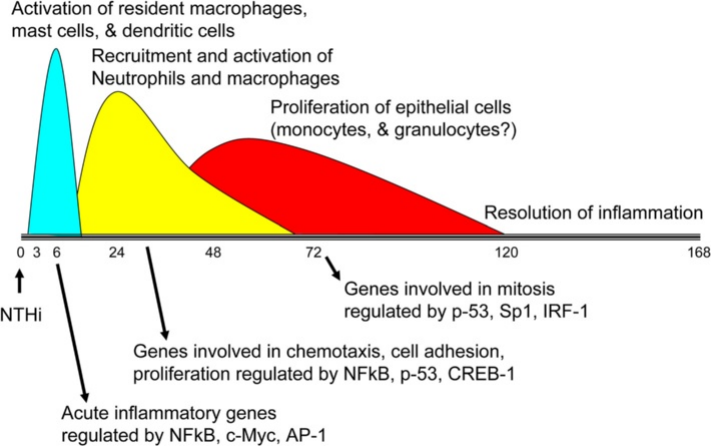
**Summary of transcriptional regulation throughout a course of acute otitis media with NTHi.** Representation of major events in the course of an acute episode of otitis media correlated with the major regulatory pathways that are associated with temporal gene clusters.

Using various systems biology approaches to integrate expression profiling with histological data, we provide a model of the changes occurring during an episode of AOM (Figure 7). The earliest gene regulation events involved activation of the innate immune and interleukin signaling networks, within hours after the mucosal epithelial cells sensed danger signals in the ME cavity. TLR activation leads to induction of NFκB and CREB target genes and the inflammatory response is initiated. Growth factors, cytokines and chemokines orchestrate the anti-microbial response and mucosal remodeling. The epithelium undergoes transition to a mesenchymal phenotype triggered by HB-EGF, and transitions from differentiated, oxidative cells to proliferative glycolytic cells allowing mucosal expansion. Neutrophils are attracted by CXCL1 and other chemoattractants produced by the epithelial cells and CSF3 promotes neutrophil survival, proliferation and differentiation which likely contribute to the histological appearance of infiltrating neutrophils starting at 6 h. Neutrophil markers are maximally induced at 24 h, that coincides with the maximum number of neutrophils by histology. Expression of NADPH oxidase subunits is maximal, mucins are produced and the alternative complement pathway is activated creating a bacteriocidal environment. Macrophages are attracted by CCL2, CCL3, CCL4 and other chemokines that are produced maximally at 24 h and macrophage markers appear at 24-72 h. Genes involved in phagocytosis are elevated between 24 and 72 h as leukocytes clear killed bacteria. Finally, genes related to tissue remodeling predominate at 5-7d post inoculation as the mucosa returns to its baseline appearance, and inflammatory cells exit the ME cavity.

Despite the extensive inflammation and the significant morphologic changes that the ME undergoes during AOM, only approximately 8% of the mouse genome transcript defines the signature of AOM with NTHi. This is a remarkably small number of genes when one considers the transformation of a simple, epithelial layer into a respiratory epithelium replete with an active inflammatory exudate. It can be speculated that, in order to preserve homeostasis, only a minimum required number of genes is activated during AOM. Furthermore, it is noteworthy that perforation of the ME bone and saline injection without NTHi induced changes in a large number of transcripts (Figure 2, Additional file 2: Figure S1), albeit at a typically much lower level of regulation than with NTHi. Thus, even in the absence of infection, injection trauma and the presence of fluid alone releases endogenous danger signals that are detectable by receptors in the ME mucosa and its resident leukocytes that work to repair the punctured mucosa and restore homeostasis. This suggests the possible existence of a common danger sensing mechanism in the ME mucosa, and underscores the importance of a sham inoculation control.

We compared our time course data with two datasets that are available in GEO, generated with different NTHi strains than the one used in the present study. MacArthur and co-workers have published a study of middle and inner ear responses to trans-tympanic heat killed NTHi versus uninjected controls (GSE49122) [40]. A single time point of 6 h post inoculation was used to assess early events. In their analysis, 2,355 genes were altered by the killed bacteria. We compared their genes with our data set from the same time point and found a 79% overlap of their significant genes with our data. We performed a similar analysis with another, unpublished dataset (GSE40087) [44] where mice received lysed NTHi by trans-tympanic injection and ME mucosae were harvested at 1 and 7d. We found 7,640 genes to be altered in their dataset by 2-way ANOVA and 63% overlap of their regulated genes with our dataset at the same post-inoculation times. Interestingly, saline injection through the tympanic membrane alone altered the expression of many genes, again indicating the importance of a sham injection control. Lastly, we compiled a list of genes that have been associated with OM in humans either through GWAS or candidate gene studies. We used GSEA to test whether any of these genes were enriched in our dataset at any time points. These OM-associated genes are significantly enriched in NTHi-inoculated mice at 3, 6, 24 and 48 h but not at later times (FDR < 0.05) (Additional file 6: Table S4). As might be expected, many of the genes are involved in the inflammatory response, for example those encoding IL-10, IL-6, IL-1A, IL-1B, CD14, FAS, MyD88, TLR2, TGFβ1, and TNF, but we also see association with EHD3, HN1, LRRC47, PALMD, PPP2R2D at early times (3-6 h), ALDH1A2, AQR, SFTPD, VLL at later times (24-48 h) and others such as COL4A2, LDLR, MUC5AC, SERPINE1, and SLFN5 that seem to be independent of time. We take this to indicate that our mouse model is representative of human disease and would be useful to test the role of these genes.

While exposure of the ME to bacteria initiates an expected pro-inflammatory response, the microarray data reveal how the ME controls and modulates this response. In addition to the rise of pro-inflammatory cytokines and their receptors early during AOM, a similarly rapid rise of anti-inflammatory interleukins such as IL-1 receptor antagonist and IL-10 is noted soon after saline or NTHi injection (Figure 6). In addition, interleukin receptors involved in inhibiting inflammatory responses, such as IL-1 type 2 receptor [45], are also significantly up-regulated with NTHi inoculation (data not shown). Finally, other negative regulators of pro-inflammatory intracellular signaling cascades, including those activated by TLRs and cytokines, are significantly up-regulated almost immediately after NTHi inoculation, highlighting how the negative regulation of inflammatory processes occurs simultaneously to pro-inflammatory processes involved in host defense.

Dysregulation of the inflammatory response has been extensively studied in the pathogenesis of various immune or inflammatory disorders, such as juvenile onset systemic rheumatoid arthritis [46] and chronic auto-inflammatory syndromes [47]. Thus an over-exuberant inflammatory response may contribute to chronic or recurrent OM in some children. However, it is equally possible that children who suffer from chronic ME disease have a blunted natural anti-inflammatory response contributing to their disease process.

There are a number of histological correlates that appear to validate our array data. The waves of gene expression patterns identified by DNA microarray analysis are corroborated by the histological findings noted after NTHi inoculation, with neutrophil-specific markers detected with the influx of neutrophils histologically into the ME, followed by macrophage-specific markers correlated with the influx of macrophages. qPCR and Western blot analyses of selected cytokines such as IL-6, IL-1 beta, TNF-alpha, and IL-10 in a murine model of AOM with NTHi in a C56BL/6 background mouse e.g. [48] exhibit similar kinetics and degrees of up-regulation to those observed in this DNA microarray data set, providing validation of the array results. However, it should be noted that the use of DNA microarrays harbors limitations for mechanistic explanations of disease processes as they only measure mRNA expression and fail to assess translation or post-translational processing of protein products, which have critical roles in determining the function of proteins. Indeed, we have recently demonstrated the importance of post-translational processing of IL-1beta by the inflammasome in OM [40].

Despite the limitations of microarrays, our array data are largely consistent with histological data [49], qPCR data [50], Western blot [51] and immunohistochemical data [52] from other animal models of NTHi-induced OM. This supports DNA microarrays or other broad gene expression assays such as NextGen sequencing, as methods for the discovery of large numbers of relevant genes throughout the course of ME disease. This application is particularly important in the *in vivo* study of AOM, as *in vivo* experiments in mouse models are especially labor intensive due to the small amount of recoverable ME mucosal tissue, making the piecewise assessment of individual genes difficult and expensive.

The transcripts up-regulated 7d after NTHi inoculation can be viewed as late resolution genes and/or “memory” genes that could prepare the ME for subsequent re-infection. In the latter category, serum amyloids are multifunctional proteins that have bacteriocidal properties [53] and serve as opsins for gram-negative bacteria [54]. The persistent up-regulation of genes related to cognate immunity suggests that the ME retains adaptive immune function after OM is resolved. This is consistent with reports that re-infection of animals with the same bacterial strain typically does not result in OM [55]. In contrast, the ME does not appear to retain innate immune “memory”. This may help to explain why the ME is so frequently subject to repeated infection, often by different strains of the same bacterium [56]. Orosomucoids are proteoglycans of uncertain function that are thought to serve as negative immune modulators involved in the resolution of inflammation [57] and may have anti-angiogenic properties [58]. Interestingly, native orosomucoids inhibit phagocytosis and killing of bacteria by PMNs and monocytes [59] but become activated by S-nitrosylation during infection to strongly inhibit bacterial growth and survival [60]. They may thus be involved in the bacteriocidal phase and the final resolution of OM.

OM in the mouse shares many of the clinical features of human ME disease caused by the same organism e.g. [11,12,21]. Obviously, generalization of animal studies to humans must be performed with caution, as there are a number of differences in the immune and inflammatory systems of mice and humans. Therefore the results of the present study must be considered with this in mind. However, it can be hoped that, because we surveyed the entire course of AOM, our data may reduce the need for animal experiments to study the expression of individual not found to be regulated, unless other evidence suggests their importance.

## Conclusion

In conclusion, transcriptome analysis serves as an exploratory step that identifies key genes and gene networks that contribute to NTHi-induced OM. In particular, the significant up-regulation of negative regulators of inflammatory responses early after NTHi inoculation, ranging from anti-inflammatory cytokines to intracellular proteins, highlights the potential regulatory mechanisms involved in the regulation of homeostasis in the ME cavity. Clearly, the ME is maintained in its normal state by a balance of pro- and anti-inflammatory signaling, as has been noted at other body sites e.g. [61]. It can be speculated that dysregulation of this balance in the ME may lead to processes such as chronic or recurrent OM in susceptible patients.

Because this method probes the responses of all genes without bias, the knowledge obtained from gene array studies may be most useful for the development of new hypotheses regarding the pathophysiology of OM, and regarding the process of OM recovery. The information derived from testing these hypotheses may ultimately afford us the possibility of developing novel treatment approaches.

### Availability of supporting data

Upon acceptance for publication, the complete raw dataset from this study will be deposited in the Gene Expression Omnibus database and appropriately referenced in the paper to assure open access.

## Additional files

Additional file 1: Table S1.
**Expression profiling data of NTHi inoculated MEs. A list of 3657 genes found to be significantly regulated by NTHi when compared to ME saline injection using at least two methods of analysis.** The table gives the probe set ID, the gene symbol, the fold change relative to untreated mice, the direction of regulation, the mean raw signal values, the RMA-corrected signals, the Entrez gene ID, the description, and the chromosomal location of genes altered during the infection. Additional file 2: Figure S1.
**A. Additional principal component analysis and down-regulated clusters. Principal component analysis of sample variation including the third principal component (treatment). Colors of symbols indicate different times and shapes of symbols indicate different treatments.** Clusters of transcripts down-regulated at 3–6 hours (B, Cluster E), 24 hours (C, Cluster F) and 3–48 hours (D, Cluster G) after inoculation. Additional file 3: Table S2.
**Enrichment analysis for gene expression clusters. For each gene cluster (genes up at 3-6 h, up at 24 h, up at 3–6 and 48–72, up at 48–72, down at 3-6 h, down at 24 h, or down at 3-48 h), worksheets report the most enriched canonical pathway maps, process networks, GO processes, transcription factor connectivity, transcription factor networks, and pathways linking to transcription factor networks.** For the pathways and processes, tables in worksheets list the total number of objects, the number of objects in the active data, the p-value and the FDR corrected p-value for the enrichment, and the network objects gene symbols involved. For the transcription factor connectivity, the table lists the actual number of connections, the expected number of connections, the ratio of actual to expected connections, the p-value and the z-score for the enrichment. For the network tables, tables show total number of nodes in the network, the number of seed nodes in active data (gene cluster), the number of pathways intersecting the network, the p-value and the z- and g-scores for enrichment. Additional file 4: Table S3.
**GSEA analysis of immunologic signatures at each post-inoculation time point. Worksheets contain the immunological signatures, the epithelial genesets, and KEGG pathways enriched at each time point following NTHi inoculation.** Tables list the size of the class, the enrichment score, the normalized enrichment score, the nominal p-value, the FDR corrected p-value (q-value), and the Bonferroni family-wise error rate corrected p-value. The top 20 signatures are presented for each time point. Additional file 5: Figure S2.
**A. An interactome analysis of PU.1-connected genes up-regulated at 24 hours after NTHi inoculation.** Genes indicated in red are significantly up-regulated. The interactome of PU.1 had the most regulated genes of any transcription factor at this time. B. Gene expression of selected genes. Expression profiles of selected cytokine, TLR and NLR gene sets. Additional file 6: Table S4. OM-associated genes enriched in NTHi-inoculated MEs by GSEA. Worksheet lists the OM-associated genes that are significantly enriched for each time point.

## Abbreviations

AOM: Acute otitis media
EMT: Epithelial to mesenchymal transition
FDR: False discovery rate
GSEA: Gene set enrichment analysis
IL-1: Interleukin-1
ME: Middle ear
MTC: Multiple testing correction
NLR: NOD-like receptor
NTHi: Non-typeable *Haemophilus influenzae*
OM: Otitis media
RMA: Robust multi-array average
TF: Transcription factor
TLR: Toll-like receptor
TNF: Tumor necrosis factor
