# Supplementary figures and images for: The transcriptome of a complete episode of acute otitis media
Source: BMC Genomics. 2015 Apr 3;16(1):259. doi: 10.1186/s12864-015-1475-7 (PMC4394589; doi:10.1186/s12864-015-1475-7)

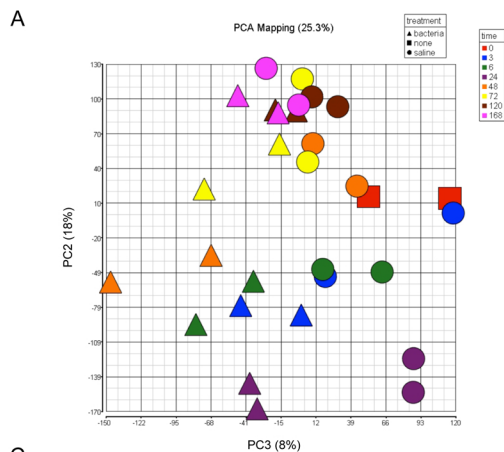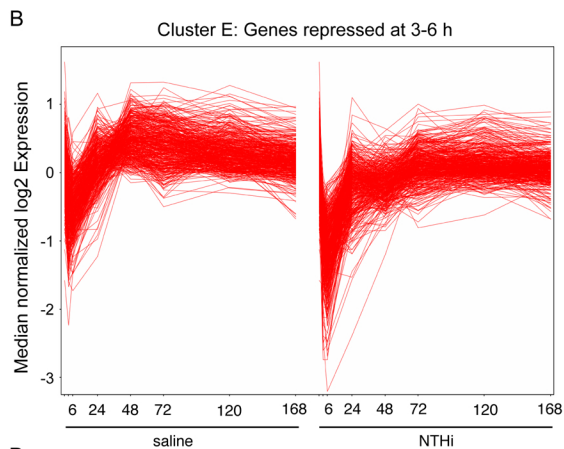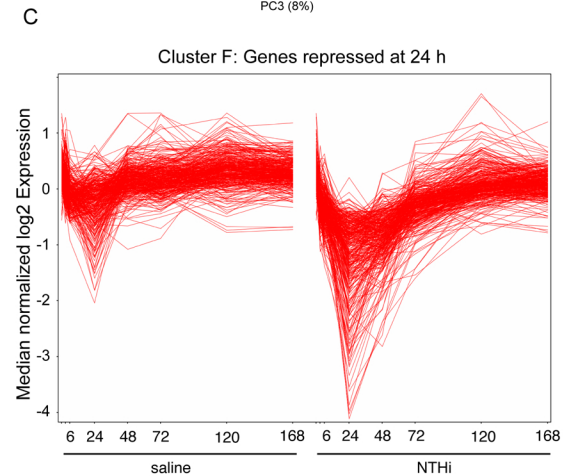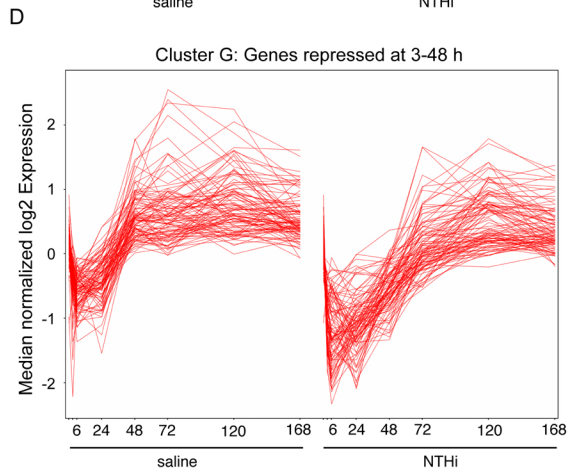

Supplement: Additional file 2: Figure S1. — A. Additional principal component analysis and down-regulated clusters. Principal component analysis of sample variation including the third principal component (treatment). Colors of symbols indicate different times and shapes of symbols indicate different treatments. Clusters of transcripts down-regulated at 3–6 hours (B, Cluster E), 24 hours (C, Cluster F) and 3–48 hours (D, Cluster G) after inoculation. [file 12864_2015_1475_MOESM2_ESM.pdf]

A

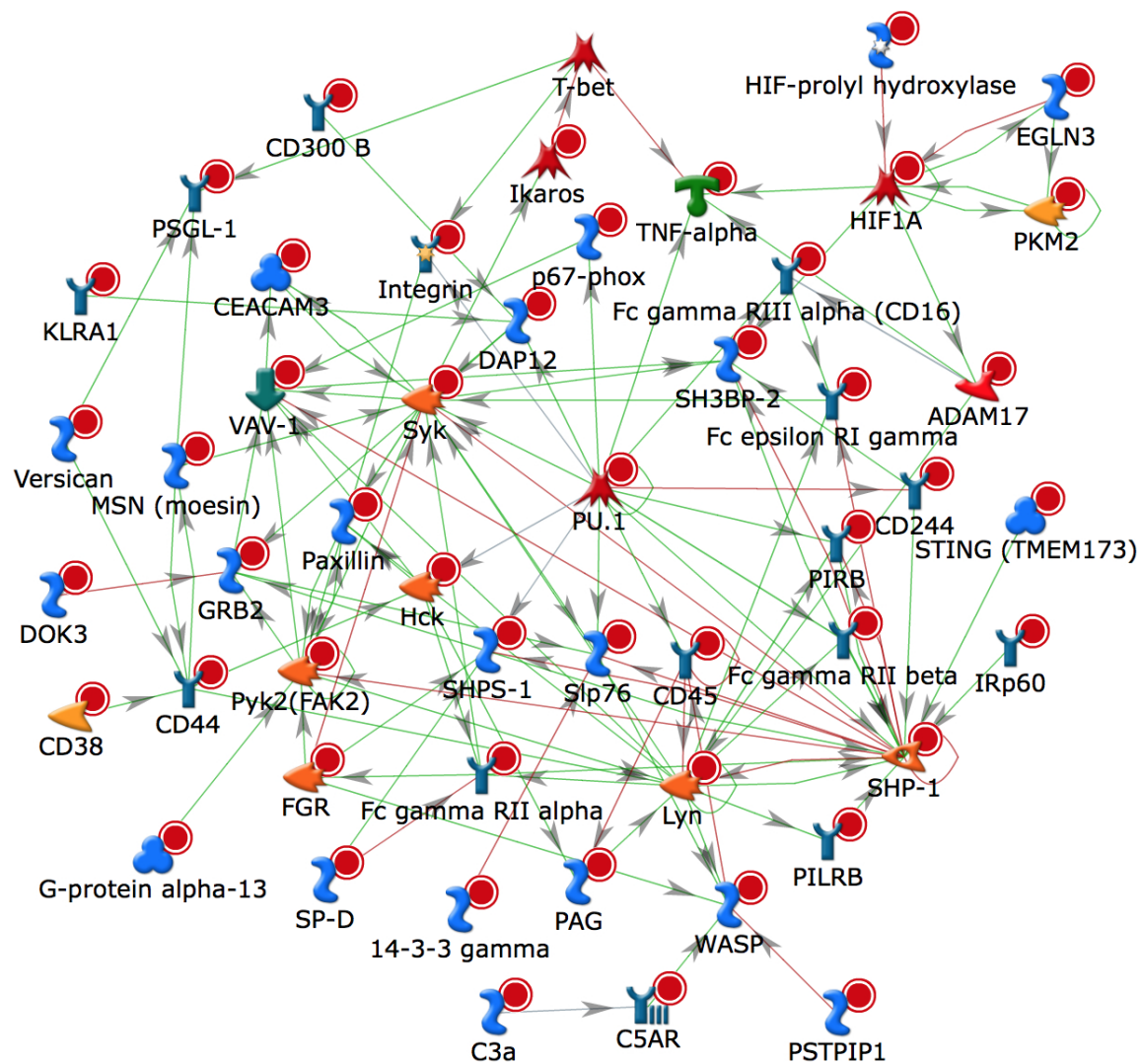

B

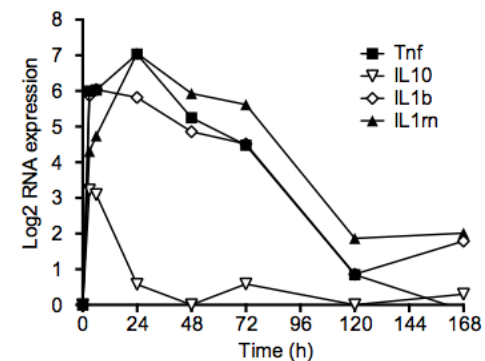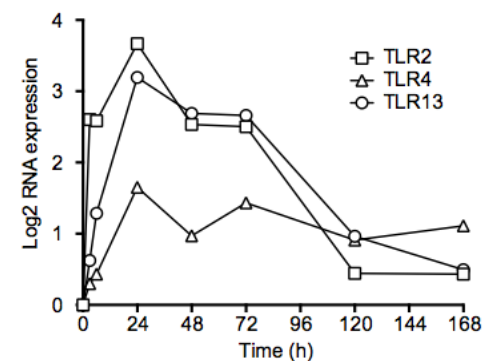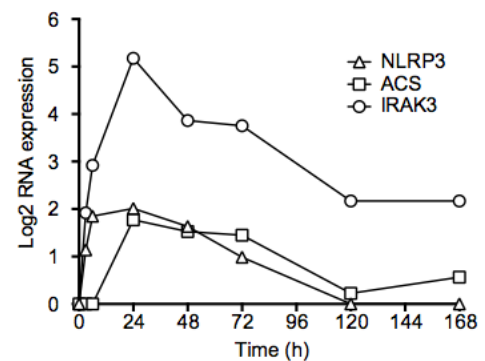

Supplement: Additional file 5: Figure S2. — A. An interactome analysis of PU.1-connected genes up-regulated at 24 hours after NTHi inoculation. Genes indicated in red are significantly up-regulated. The interactome of PU.1 had the most regulated genes of any transcription factor at this time. B. Gene expression of selected genes. Expression profiles of selected cytokine, TLR and NLR gene sets. [file 12864_2015_1475_MOESM5_ESM.pdf]
